# Supplementation with Omega-3 Fatty Acids in Psychiatric Disorders: A Review of Literature Data

**DOI:** 10.3390/jcm5080067

**Published:** 2016-07-27

**Authors:** Paola Bozzatello, Elena Brignolo, Elisa De Grandi, Silvio Bellino

**Affiliations:** Centre for Personality Disorders, Department of Neuroscience, University of Turin, 10126 Turin, Italy; paola.bozzatello@unito.it (P.B.); elena.brignolo@yahoo.com (E.B.); elisa.degrandi@gmail.com (E.D.G.)

**Keywords:** polyunsaturated fatty acids, omega-3 fatty acids, mood disorders, schizophrenia, borderline personality disorder, attention deficit hyperactivity disorder, eating disorders, substance use disorder, tolerability

## Abstract

A new application for omega-3 fatty acids has recently emerged, concerning the treatment of several mental disorders. This indication is supported by data of neurobiological research, as highly unsaturated fatty acids (HUFAs) are highly concentrated in neural phospholipids and are important components of the neuronal cell membrane. They modulate the mechanisms of brain cell signaling, including the dopaminergic and serotonergic pathways. The aim of this review is to provide a complete and updated account of the empirical evidence of the efficacy and safety that are currently available for omega-3 fatty acids in the treatment of psychiatric disorders. The main evidence for the effectiveness of eicosapentaenoic acid (EPA) and docosahexaenoic acid (DHA) has been obtained in mood disorders, in particular in the treatment of depressive symptoms in unipolar and bipolar depression. There is some evidence to support the use of omega-3 fatty acids in the treatment of conditions characterized by a high level of impulsivity and aggression and borderline personality disorders. In patients with attention deficit hyperactivity disorder, small-to-modest effects of omega-3 HUFAs have been found. The most promising results have been reported by studies using high doses of EPA or the association of omega-3 and omega-6 fatty acids. In schizophrenia, current data are not conclusive and do not allow us either to refuse or support the indication of omega-3 fatty acids. For the remaining psychiatric disturbances, including autism spectrum disorders, anxiety disorders, obsessive-compulsive disorder, eating disorders and substance use disorder, the data are too scarce to draw any conclusion. Concerning tolerability, several studies concluded that omega-3 can be considered safe and well tolerated at doses up to 5 g/day.

## 1. Introduction

The role of omega-3 highly unsaturated fatty acids (HUFAs) in human mental health has been widely studied in the last two decades.

Omega-3 fatty acids eicosapentaenoic acid (EPA) and docosahexaenoic acid (DHA) are derived from alpha-linolenic acid (ALA) and are dietary essential fatty acids. They cannot be synthetized de novo by mammals and are provided by supplementation, such as fish oil. 

EPA and DHA act as competitive inhibitors of omega-6 fatty acids causing a reduction in the synthesis of pro-inflammatory mediators [1]. In fact, the omega-6 family of fatty acids is converted to arachidonic acid and then into prostaglandins and leukotrienes, which are responsible of the pro-inflammatory effects. Therefore, a diet rich in fish oil has been shown to decrease the incidence of inflammatory diseases.

In addition, HUFAs slow coronary atherosclerosis by optimizing cholesterol concentrations and lowering plasma triglyceride levels. HUFAs also have antithrombotic, antiarrhythmic and vasodilatory properties, providing the protection of the cardiovascular system and significantly diminishing cardiovascular mortality. These effects of omega-3 fatty acids have supported indications in the secondary prevention of hypertension, coronary heart disease, type 2 diabetes and in some cases of rheumatoid arthritis, Crohn’s disease, ulcerative colitis, chronic obstructive pulmonary disease and renal disease [2,3].

EPA and DHA are important for fetal development, including neuronal, retinal and immune functions [4].

In recent years, the interest of omega-3 fatty acids has grown in psychiatry, and their role in the treatment of several mental diseases has been investigated. HUFAs are important components of phospholipids and cholesterol esters of the neuronal cell membrane, especially of dendritic and synaptic membranes. The rationale for the use of these new agents in psychiatric disorders stemmed from their primary action in producing modification of the synaptic membrane [5]. Indeed, they modulate and are involved in brain cell signaling, including monoamine regulation [6,7,8,9,10], modification of receptor properties or the activation of signal transduction by receptors.

Among psychiatric diseases, long-chain omega-3 fatty acids have been tested in the treatment of schizophrenia, unipolar and bipolar mood disorders, anxiety disorders, obsessive-compulsive disorder, attention deficit hyperactivity disorder (ADHD), autism, aggression, hostility and impulsivity, borderline personality disorder, substance abuse and anorexia nervosa. The aim of this review is to provide an updated account of the empirical evidence of the efficacy and safety that are currently available for omega-3 fatty acids in the treatment of mental disorders. In particular, we focused on the data of efficacy and the adverse effects of different doses of HUFAs in the treatment of mental disorders, to determine which dose levels of single or combined fatty acids are related to a good clinical response of specific mental disorders with a low risk of significant adverse effects. 

## 2. Methods

This review presents and critically evaluates data from clinical trials, systematic reviews and meta-analyses published from 1980 to September 2015, after a systematic research on the Medline database provided by the U.S. National Library of Medicine (http://www.ncbi.nlm.nih.gov/pubmed?otool=iitutolib). The search terms were: omega-3 fatty acids; HUFAs; eicosapentaenoic acid; docosahexaenoic acid; ethyl-eicosapentaenoic acid; α-lipoic acid; α-linoleic acid; psychiatric disorders; psychotic disorders; schizophrenia; mood disorders; anxiety disorders; obsessive-compulsive disorder; attention deficit hyperactivity disorder; autism spectrum disorders; aggression, hostility, impulsivity; personality disorders; borderline personality disorder; substance dependence; anorexia nervosa; adverse effects; dose.

### 2.1. Schizophrenia

Studies of post mortem samples have shown that patients with schizophrenia have often low levels of EPA and DHA in their brain cells. The level of fatty acids is considered important to maintain the correct nerve cell-membrane metabolism [11]. The evidence is strong enough to have led to a ‘membrane phospholipid hypothesis’ of schizophrenia [12,13]. Based on findings that in individuals with schizophrenia or related psychotic disorders, certain omega-3 and omega-6 HUFA levels are reduced compared to healthy control samples, the idea that restoration of HUFA resources could be used as a treatment option in psychotic disorders has been widely discussed [14].

To date, there are 13 RCTs available concerning the role of omega-3 fatty acids supplementation in schizophrenia or related disorders. For ethical and clinical reasons, most trials used the add-on strategy, including patients who already received neuroleptics or atypical antipsychotics. Two exceptions are the studies published by Amminger [15] and Markulev [16], which considered patients at high risk for developing psychosis. Peet et al. [17] and Emsley et al. [18] designed trials with omega-3 HUFAs as a monotherapy, but in both cases, almost all patients needed to be treated also with an antipsychotic drug during the course of the trial. Only six RCTs [15,17,19,20,21,22] reported that HUFAs had a benefit on positive or negative symptoms. The sample size of all of the reviewed studies was small, and the population investigated suffered from a considerable degree of heterogeneity. In fact, not only patients with a diagnosis of schizophrenia were included, but also subjects with schizoaffective disorder [23], first episode of psychosis [21,24] and resistant schizophrenia [19]. The omega-3 supplementation has been administered in a dosage ranging from 1–4 g/day. The duration of the trials has been limited to 8–16 weeks in the majority of the studies. The main issue is that no conclusions could be drawn regarding the medium- to long-term effects of HUFAs in schizophrenia, according to the findings of the four meta-analysis available on this topic [25,26,27,28] (Table 1).

Peet and colleagues [17] performed a 12-week placebo-controlled trial in 30 males and 15 females on stable antipsychotic medication who were still symptomatic. Only 35 patients completed the trial. The authors found that EPA was more effective at the reduction of symptoms as assessed with the Positive and Negative Syndrome Scale (PANSS) in comparison to DHA and placebo. In the same year, Peet and colleagues [17] conducted a second RCT to test EPA (dose: 2 g/day) as a monotherapy for schizophrenia. Thirty patients who never assumed an antipsychotic agent with a recent diagnosis of schizophrenia were recruited. For ethical reasons, antipsychotic medications were permitted, so at the end of the study, only two patients remained without an antipsychotic treatment. The results indicated that patients treated with EPA, but also antipsychotics, improved more than placebo-treated subjects in psychopathology measured with the PANSS. In 2002, Peet and Horrobin [19] administered ethyl-EPA to patients with resistant schizophrenia for 12 weeks. One hundred and fifteen subjects, who were already in treatment with clozapine, olanzapine, risperidone, quetiapine or a neuroleptic, were randomly assigned to placebo or 1, 2 or 4 g/day of ethyl-EPA arm in addition to antipsychotic drugs. Authors reported a significant improvement of the PANSS total score and subscales scores in the 2 g/day EPA-treated group, but there was also a large effect of placebo in patients receiving only typical or new generation antipsychotics. In contrast, in patients on clozapine, there was little placebo response, but a significant effect of augmentation with ethyl-EPA on all rating scales, PANSS, PANSS subscales and the Montgomery-Asberg Depression Rating Scale (MADRS).

More recently, Jamilian and colleagues [20] compared 1 g/day of non-specified omega-3 with placebo in 60 patients with schizophrenia who already assumed a standard antipsychotic medication. Omega-3 outperformed placebo significantly, based on the PANSS score.

Less encouraging findings were presented by Fenton and colleagues [23] who designed a 16-week study to evaluate the efficacy of ethyl-EPA (3 g/day) vs. placebo in 87 patients with schizophrenia or schizoaffective disorder with residual symptoms in treatment with conventional antipsychotics. The results indicated no significant differences in positive or negative symptoms, mood and cognition. Similar results were reported by Berger and colleagues [24], who performed an RCT including 69 patients with first-episode psychosis who were treated for 12 weeks with a flexible dose of antipsychotic medication (risperidone, olanzapine or quetiapine) plus ethyl-EPA (E-EPA) (2 g/day) or placebo. The authors suggested that E-EPA may accelerate treatment response and ameliorate the tolerability of antipsychotics, but they remained skeptical about the specific efficacy of EPA in early psychosis. 

Early treatment of the prodromal period of psychotic disorders has been linked to more favorable outcomes. The term ‘ultra-high-risk’ (UHR) identifies individuals who are at increased risk of developing full blown psychotic symptoms. Treatment with omega-3 HUFAs may be an interesting treatment option for UHR subjects due to the low incidence of adverse effects. 

Amminger and colleagues [15] performed an RCT on 76 individuals with high risk for developing psychosis comparing omega-3 HUFAs (1.2 g/day) to placebo during a period of 12 weeks. This phase was followed by a further 40-week monitoring period. This study showed that fatty acids may prevent the progression in psychosis in young UHR patients. Recently, in a multicenter double-blind randomized study, 304 participants meeting the ‘at-risk’ criteria were allocated to treatment with either omega-3 plus cognitive-behavioral case management (CBCM) or placebo plus CBCM. The total length of treatment was six months. The data collected are still under evaluation [16].

Another recent RCT was designed by Pawelczyk et al. [21] to compare the efficacy of 2.2 g/day of HUFAs or olive oil placebo added on to an antipsychotic medication with regard to symptom severity in patients suffering from first-episode schizophrenia. An improvement in psychopathology and the level of functioning was observed.

In the last few years, the first placebo-controlled trial on the association of omega-3 fatty acid and redox regulators for treating schizophrenia was conducted for 16 weeks by Bentsen and colleagues [22]. The participants were 99 patients with a diagnosis of schizophrenia or related psychoses who were divided in four groups (double placebo, active vitamins, active EPA or double active). The psychotic symptoms were assessed with PANSS. The results showed that adding E-EPA 2 g/day or vitamin E 364 mg·day/1 plus vitamin C 1000 mg/day to antipsychotic drugs prevented the course of psychosis, but only among patients with low levels of erythrocyte HUFAs. On the contrary, combining EPA and the vitamins neutralized this detrimental effect. Emsley and colleagues [18] tested a combination of omega-3 polyunsaturated fatty acids (EPA 2 g/day and DHA 1 g/day) and a metabolic antioxidant, alpha-lipoic acid (α-LA 300 mg/day), as prevention treatment of relapse after antipsychotic discontinuation in subjects who were successfully treated for 2–3 years after a first-episode of schizophrenia or schizoaffective disorder. Unfortunately, this study was affected by a small sample size and by premature termination of recruitment due to the high relapse rates and the severity of the relapse episodes. Finally, the results failed to support evidence that HUFAs + α-LA could be a worthwhile tool to maintenance antipsychotic treatment in relapse prevention.

Emsley et al. [29,30] also investigated the efficacy of long-chain fatty acids in reducing side effects due to conventional antipsychotic treatment in two randomized controlled trials. In particular, they verified the effects of omega-3 fatty acids supplementation on extrapyramidal symptoms. The first study [29] found some beneficial effects of E-EPA (3 g/day) as add-on treatment in 40 patients with chronic schizophrenia, who had received a stable antipsychotic medication for at least six months on extrapyramidal symptoms assessed with the Extrapyramidal Symptoms Rating Scale. Moreover, the authors in this study also found a significant improvement of positive and negative symptoms measured with the PANSS. Unfortunately, the second study [30], disconfirmed these results in a larger sample of 77 patients with schizophrenia or schizoaffective disorders and tardive dyskinesia. In this trial, patients received E-EPA (2 g/day) or placebo for 12 weeks. The E-EPA arm had an initial improvement in dyskinesia, but this result was not stable beyond six weeks. 

Four meta-analyses [25,26,27,28] of RCTs were conducted in order to establish if the available data support the use of omega-3 fatty acids in patients affected by schizophrenia or other psychotic disorders. The authors concluded that the evidence in favor of omega-3s as psychotropic agents in schizophrenia is preliminary, and the findings remain yet inconclusive. 

### 2.2. Major Depressive Disorder

In 1998, Hibbeln [31] discovered a direct and powerful inverse association between fish consumption and the prevalence of major depression in a study aimed to test the hypothesis that a high consumption of fish could be correlated with a lower annual prevalence of major depression. One year later, it was established that depression often co-occurs with cardiovascular disease, which is associated with elevated cholesterol and lower fatty acids plasma levels [32]. After this discovery, abnormal fatty acid compositions in the peripheral tissues (e.g., plasma, serum and red blood cells) of patients with depression have been reported extensively [33,34].

Twenty randomized controlled trials were conducted in order to evaluate the effectiveness of omega-3, EPA and DHA, in the treatment of mild or moderate depressive disorder. Fatty acids were administered both as a monotherapy and as supplementation to ongoing pharmacotherapy or psychotherapy. Doses ranged from 0.4–4.4 g/day of EPA and from 0.2–2.4 g/day of DHA. Ten RCTs investigated the efficacy of omega-3 fatty acids as an individual treatment strategy, but only seven of them reported that EPA and/or DHA had a positive effect on core depressive symptoms. Seven RCTs aimed to determine whether administering omega-3 fatty acids provides any additional benefit to conventional patient treatment (e.g., fluoxetine, citalopram or sertraline) for major depression. Five of these studies obtained a significant improvement of depressive symptoms [35,36,37,38,39]. However, the validity of these findings is limited by the heterogeneity of methods among different studies, including the use of unstandardized assessment instruments of depressive symptoms, as well as considerable differences in doses and ratios of omega-3 fatty acids, the duration of the trials and the demographic and clinical characteristics of the samples. Further studies are needed to better understand the mechanisms of the antidepressant effects of HUFAs and to explain the reason for the discordant results published until now.

Peet and Horrobin [35] conducted a 12-week study that showed improvement in patients treated with 1 g/day of ethyl-EPA (E-EPA) and who were refractory to selective serotonin uptake inhibitor monotherapy for major depression. Findings showed that only 1 g/day of omega-3, but not 2 or 4 g/day, was effective in reducing depressive symptoms measured with the Hamilton Depression Rating Scale (HDRS), the MADRS and the Back Depression Inventory (BDI) (effect size: 0.92) in adults with ongoing depression. Nemets et al. [40] designed a four-week placebo-controlled study on 20 subjects already undergoing antidepressant therapy and found better outcome results in the treatment group with E-EPA at the dosage of 2 g/day. The effect of omega-3 was significant from the second week of treatment, similarly to the time of response to antidepressants and resulting in an improvement of core depressive symptoms on the HDRS. The same authors [41] suggested that omega-3 may have a therapeutic benefit in 6–12-year-old children with major depression. Of the 20 patients who entered data analysis, 10 received placebo and 10 received omega-3 (0.4 g/day of EPA plus 0.2 g/day of DHA) for 16 weeks. Depression symptoms were assessed with the Childhood Depression Rating Scale, the Childhood Depression Inventory and the Clinical Global Impression (CGI). Su and colleagues [36] conducted an eight-week RCT comparing EPA (at the dose of 4.4 g/day) plus DHA (at the dose of 2.2 g/day) with placebo in augmentation to antidepressant in 22 patients with major depression. They found encouraging results: participants who were treated with omega-3 fatty acids had a significantly lower score of the HDRS. An interesting study on 36 pregnant women with major depressive disorder [42] compared omega-3 HUFAs monotherapy (2.2 g/day of EPA plus 1.2 g/day of DHA) with placebo. Twenty-four patients who finished the trial showed significantly lower depressive symptoms’ ratings on the HDRS, the Edinburgh Postnatal Depression Scale (EPDS), and the BDI. The findings of this study are remarkable because omega-3s are preferentially transported to the growing fetus during pregnancy, which can deplete fatty acid levels in mothers. 

The beneficial effect of omega-3 fatty acids supplementation on depressive symptoms was also confirmed in elderly women (aged 66–95 years) residents in a nursing home [43,44]. Within a context of an eight-week double-blind RCT, 46 women who received a diagnosis of depression were randomly assigned to the intervention group (1.67 g/day of EPA plus 0.83 g/day of DHA) or the placebo group. Depressive symptoms, assessed with the Geriatric Depression Scale, quality of life assessed with the Short-Form 36-Item Health Survey and phospholipids fatty acids’ profile improved. Nevertheless, the same authors in another study [45] measured some immunological parameters and cytokines in the same sample, but they did not find a significant amelioration of the immunological function in the intervention group.

Currently, there is not a general consensus concerning the efficacy of omega-3s in the treatment of depression. Rees and colleagues [46] found that omega-3 fatty acids were not superior over placebo in treating perinatal depressive symptoms. They administered 0.4 g/day of EPA plus 1.6 g/day of DHA as a monotherapy to 26 pregnant women for six weeks. Similar results were collected by Freeman and colleagues [47], who did not detect significant differences between omega-3 HUFAs (EPA 1.1 g/day plus DHA at the dose of 0.8 g/day) and placebo on perinatal depressive symptoms measured with the HDRS and the EPDS. All 59 women enrolled in this trial received also a supportive psychotherapy for eight weeks.

In a similar way, omega-3s were not found superior to placebo in treating depressive symptoms in three other studies. Marangell et al. [48], Silvers et al. [49] and Grenyer et al. [50] obtained no significant improvement in the symptoms of depression in patients who were treated with similar doses of EPA and/or DHA (ranging 2–3 g/day) as sole therapy or in addition to antidepressive drug. These studies were different for sample size, duration and instruments to assess depressive symptoms, but they drew the same conclusions.

Lespérance et al. (2011) [37] conducted an inclusive, double-blind, randomized controlled trial on 432 patients with a major depressive episode. They administered EPA (1.050 g/day) and DHA (150 mg/day) for eight weeks to a sample with a heterogeneous therapy (40.3% of the patients already received an antidepressant at the baseline). Omega-3 supplementation resulted in being superior over placebo only for the patients without comorbid anxiety disorders. 

Two studies investigated the effectiveness of omega-3s on depression and on cognitive functions. Rogers et al. [51] in a 12-week trial tested the efficacy of EPA plus DHA (0.6 g/day + 0.85 g/day) on mood and cognition in 218 patients with mild to moderate depression. More recently, Antypa and colleagues [52] treated 71 depressed patients with omega-3 fatty acids or placebo for four weeks. They evaluated cognitive reactivity with the Leiden Index of Depression Sensitivity-Revised (LEIDS-R) and the Profile of Mood States (POMS), and depressive symptoms were measured with the BDI. Both studies have not obtained favorable results on mood and cognition using omega-3s.

Two trials compared the effectiveness of different doses of EPA or DHA in the treatment of depressive disorders. Mischoulon and colleagues in 2008 [53] enrolled 35 depressed outpatients that were randomized into one of three double-blind dosing arms for 12 weeks: 1 g/day DHA; 2 g/day DHA; 4 g/day DHA. In this study, subjects who received 2 g/day or more DHA had a lower response rate (on the HDRS) compared to those who received 1 g/day. It is interesting to note that these findings are similar to Peet and Horrobin’s results [35]: E-EPA, as well as DHA appear more effective at lower doses. In the same year (2008), Jazayeri and colleagues [38] compared EPA at the low dosage of 1 g/day with fluoxetine (20 mg/day) in 60 patients with major depressive disorder for eight weeks. The results showed that a low dose of EPA may have similar therapeutic effects as fluoxetine.

A recent and larger eight-week RCT [54] compared the potential therapeutic effects of three differing doses, (i) EPA (1 g/day), (ii) DHA (1 g/day) or (iii) placebo (containing 980 mg of soybean oil per cap; four capsules/day), in 196 depressed patients. The authors reported a significant improvement in depressive symptoms in all three groups; neither EPA nor DHA were superior to placebo.

Interesting results were obtained from one study that explored the association of omega-3 fatty acids and antidepressants in treating depressed patients [39]. The authors investigated the efficacy of combination therapy with citalopram plus omega-3 fatty acids (1.8 g/day of EPA + 0.4 g/day of DHA + 0.2 g/day of other omega-3s) vs. citalopram plus placebo in 42 patients with initial depression. Combined therapy showed grater improvements in depression symptoms assessed with HDRS, but did not enhance the speed of antidepressant response.

The effects of fish oil supplementation were also investigated in patients with major depression associated with coronary heart disease [55]. Carney and colleagues (2009) obtained negative results testing omega-3 in cardiopathic patients. They performed a 10-week RCT to evaluate the effects of ethyl-EPA (0.93 g/day) plus DHA (0.75 g/day) in addition to sertraline on symptoms of depression in 122 patients with major depressive disorder and coronary heart disease. The authors did not find any superiority of treatment over placebo.

In conclusion, supplementation with HUFA in patients with major depression seems useful in improving depressive symptoms, but the findings are not univocal. Systematic reviews and meta-analyses also provided controversial conclusions. Appleton and colleagues [56] reviewed 35 RCTs that included 329 adults and concluded that trials investigating the effects of HUFAs on depression are increasing, but it is difficult to evaluate their results because of data heterogeneity. Bloch and Hannestad [57] outlined the methodological limits of available studies that affect the validity of results. On the contrary, other meta-analyses [58,59,60] indicated a more encouraging prospective in this field and concluded that omega-3 fatty acids produce a significant antidepressant effect. In particular, EPA seems to be more efficacious than DHA in treating depression. A more recent comprehensive meta-analysis [61] of RCTs confirmed that the use of omega-3s as therapeutic agents was effective in patients with the diagnosis of major depression or depressive symptoms and suggested that patients with greater depressive severity and those meeting full criteria for a diagnosis of depressive disorder demonstrated greater treatment gains (Table 2).

### 2.3. Bipolar Disorders

In patients with a diagnosis of bipolar disorder, lower erythrocyte membrane levels of omega-3 fatty acids have been observed [62]. Based on this consideration, several investigators performed clinical trials to evaluate whether the implementation of these agents may impact the clinical features of this disorder. To date, only seven RCTs on omega-3 treatment for bipolar disorders have been published. Except for the first pioneering trial, they were all augmentation studies. Intervention length ranged from 6–16 weeks. Three of these [63,64,65] used EPA in addition to stable psychotropic medication. Two studies [66,67] explored the combination of EPA and DHA in addition to mood stabilizers or other usual treatments. In one trial [68], alpha-linolenic acid was administered in association with stable psychotropic medications. Finally, in one add-on RCT [69], omega-3 was associated with cytidine or not as augmentation to a stable medication. Doses ranged from 0.3–6.2 g/day of EPA and from 0.2–3.4 g/day of DHA. The dose of alpha-linolenic acid was 6.6 g/day.

In the first trial, Stoll and colleagues [66] administered a combination of EPA (6.2 g/day) and DHA (3.4 g/day) as a monotherapy to 14 patients, while 16 patients received placebo for 16 weeks. This study was affected by a large number (13 subjects) of drop-outs because of depression, mania, hypomania and mixed state. Outcomes revealed the efficacy of omega-3 in terms of the reduction and remission of depressive symptoms (HDRS), whereas no significant effects were registered on mania (Young Mania Rating Scale (YMRS)). These findings were replicated by Frangou and colleagues [63] in a 12-week controlled study including 75 patients treated with ethyl-EPA (1 or 2 g/day) as adjunctive treatment to stable psychotropic medications. The same authors [64] reported increased brain levels of *N*-acetyl-aspartate (NAA), a presumed marker of neuronal integrity, with 2 g/day of ethyl-EPA in 14 female patients with type I bipolar disorder that were treated with a stable dose of lithium. The study was controlled with placebo. The rise in NAA level provided the first evidence for a neurotrophic role of E-EPA treatment in bipolar disorder. 

Concerning the manic phase, less encouraging findings were presented by Chiu et al. [67], who designed a four-week study to evaluate the efficacy of 4.4 g/day of EPA plus 2.4 g/day of DHA vs. placebo in addition to a fixed dose of valproate (20 mg/kg/day) in 15 patients with acute mania. The authors found a reduction in both groups of the YMRS scores from baseline, with no significant differences between omega-3 and placebo. Similar results were obtained in a larger RCT [65], which involved 121 patients with bipolar depression or rapid cycling bipolar disorder. The adjunction of 6 g/day of EPA to at least one mood stabilizer did not produce meaningful differences in reducing the severity of depressive or manic symptoms, assessed respectively with the Inventory for Depressive Symptomology total and the YMRS.

Gracious and colleagues [68] conducted a double-blind RCT of alpha-linoleic acid (ALA) as flax seed oil in children and adolescents with bipolar I or II disorder, assuming a stable psychotropic medication. In this study, there was a high rate of noncompliance with taking supplements because of the large number of capsules required (up to 12 per day). Those who were treated with an adequate amount of ALA, demonstrated a significant improvement of overall symptom severity in comparison with the placebo group. Nevertheless, depression and mania measures did not show significant differences between treatment and placebo groups. More recently, therapeutic properties of omega-3 fatty acids in bipolar disorder were further denied by Murphy et al. [69] in a four-month RCT. Forty-five patients with a diagnosis of type I bipolar disorder, who were already treated with a mood stabilizer, were randomly assigned to three different groups consisting of omega-3 fatty acids plus cytidine, omega-3 fatty acid plus placebo or only placebo. In this study, supplementation with omega-3 did not produce a significant improvement in affective symptoms over an extended period of treatment. 

Overall, a moderate antidepressant effect was observed for adjunctive omega-3 agents compared to conventional therapy alone in the treatment of bipolar depression [66,67]. The small number of studies, the heterogeneity of HUFA doses and ratios and the small sample sizes were important limitations to obtain reliable data on this topic. 

In summary, some beneficial effects of omega-3 HUFAs in bipolar disorders were observed. The conclusions of systematic reviews and meta-analyses [70,71,72,73] provided initial evidence that bipolar depressive symptoms, but not manic symptoms, may be improved by adjunctive administration of omega-3 fatty acids (Table 3).

### 2.4. Anxiety Disorders

The hypothesis that omega-3s may have anxiolytic properties was formulated in the light of the frequent comorbidity between mood disorders and anxiety disorders and the effectiveness of some conventional pharmacotherapy on both disorders. Furthermore, low omega-3 erythrocyte membrane levels have been observed in patients with anxiety disorders [74,75,76]. Unfortunately, to our knowledge, there are no RCTs that have systematically investigated the effect of omega-3 PUFA in anxiety disorders. Only a small trial [77] showed a reduction in the levels of anxiety and tension in 24 substance abusers. Participants received 2.2 g of EPA/day plus 0.5 g of DHA/day vs. placebo (vegetable oil) for three months. Anxiety continued to be significantly decreased in the active treatment group after three months’ discontinuation. In each case, the evidence supporting the use of omega-3 PUFAs as an anxiolytic is currently insufficient [78]. 

### 2.5. Obsessive-Compulsive Disorder

Only one RCT about obsessive-compulsive disorder was published by Fux and colleagues [79], who administered 2 g/day of EPA or placebo in augmentation of a stable dose of SSRIs to 11 patients (four of these received 40 mg; two received 30 mg; and two received 60 mg of paroxetine daily; one patient was on 250 mg of fluvoxamine daily; and two were on 40 mg of fluoxetine daily) for six weeks. The augmentation with HUFAs was not associated with significant improvement of anxious, obsessive-compulsive and depressive symptoms compared to placebo.

### 2.6. Attention Deficit Hyperactivity Disorder

Increasing attention has been given to the role of HUFAs in childhood developmental disorders. Since the 1980s, reduced HUFA levels have been reported in blood analysis of hyperactive children compared to healthy controls [80,81,82,83]. Recently, several double-blind placebo controlled trials have been conducted to assess the efficacy of omega-3 fatty acid in the treatment of children with ADHD [84,85,86,87,88,89,90,91,92,93,94,95], but results are still discordant and disputable. Due to the considerable heterogeneity of published investigations, additional large-cohort studies and well-designed clinical trials of ADHD patients are required. In particular, studies should be conducted with strict criteria concerning methodological issues, such as an accurate DSM-5 diagnosis of ADHD, a double-blind controlled design, the choice of reliable assessment instruments of symptoms and functioning, a clear definition of the doses and ratios of omega-3 fatty acids and of the possible association with conventional medications. 

If we examine the available data, seven [84,85,87,88,89,90] of thirteen RCTs investigating the efficacy of omega-3 fatty acids had a positive effect on ADHD-related symptoms. The remaining studies did not obtain significant improvement in ADHD symptoms [81,91,92,93,94,95]. Only two of these thirteen RCTs aimed to determine whether taking omega-3 fatty acids confers any additional benefit to conventional patient treatment (stimulant and non-stimulant medications): one found negative results [91] and one positive [90]. 

In particular, not very encouraging findings have been presented by six RCTs comparing the association of EPA and DHA to placebo. In a double-blind study performed by Stevens and colleagues [81], fifty patients with ADHD-like symptoms received either placebo (olive oil) or HUFA supplementation (480 mg of DHA, 80 mg of EPA, 40 mg of arachidonic acid and 96 mg of gamma-linolenic acid/day) for four months. At baseline and at the end of the intervention period, both parents and teachers completed the Conners’ Abbreviated Symptom Questionnaires (ASQ) and the Disruptive Behavior Disorders (DBD) Rating Scale. Other additional neuropsychological tests were administered: the Conners’ Continuous Performance Test (CPT) and eight tests of cognitive ability of the Woodcock-Johnson Psycho-Educational Battery-Revised (WJ-R). No significant difference between active group and placebo was observed for any rating scale comparing patients who completed the trial. HUFAs supplementation led to a significant behavioral improvement over placebo on only two of the 16 outcome measures that were used (DBD-Conduct for Parents and the DBD-Attention for Teachers) and by intention-to-treat analysis only.

In 2004, Hirayama and colleagues [92] also reported no evidence of efficacy of omega-3 fatty acids compared to placebo in treating ADHD symptoms. In this double-blind placebo controlled trial, the majority of the 40 children did not receive ADHD medications (only six of them had been under conventional medications). They administered both DHA and EPA through fish oil-enriched food (the daily dose was approximately 100 mg of EPA and 500 mg of DHA) to twenty children with ADHD for two months. The control group (*n* = 20) took indistinguishable food without fish oil. The authors measured ADHD related symptoms according to the DSM-IV criteria, aggression, impatience and some cognitive features, but they did not find any significant changes in outcome measures. 

In another study (Johnson and colleagues) [93], seventy-five children and adolescents 8–18 years old with ADHD were included and treated with 558 mg EPA, 174 mg DHA and 60 mg gamma linoleic acid daily, compared to placebo. Only one of the patients had been previously treated with a conventional drug for ADHD (methylphenidate). Investigators found that only a subgroup of patients characterized by the inattentive subtype of ADHD and associated neurodevelopmental disorders showed a meaningful clinical response to omega-3 and omega-6 treatment. They concluded the study results were essentially negative and did not support the superiority of HUFAs over placebo.

Milte [94] performed a double-blind RCT, including 90 children 7–12 years old with ADHD treated with EPA-rich oil (providing 1109 mg of EPA and 108 mg of DHA), DHA-rich oil (providing 264 mg of EPA and 1032 mg of DHA) vs. an omega-6 HUFA oil during a period of four months. Children were taking no other medication. Despite that this study demonstrated no statistically-significant differences between the two groups, the authors found that increased levels of erythrocyte DHA seemed associated with improved word reading and lower parent ratings of oppositional behavior. Interestingly, a subgroup of 17 patients with learning difficulties exhibited superior benefits from the supplementation with omega-3 fatty acids.

A more recent randomized, double-blind controlled trial (Widenhorn-Müller et al.) [95] was conducted in 95 ADHD patients aged between six and 12 years who received omega-3 fatty acids or placebo for 16 weeks, not treated with medications for ADHD. The authors found less negative results than those from previous studies, but not yet satisfactory. Supplementation with EPA and DHA (600 mg of EPA and 120 mg of DHA daily) improved working memory function, but had no effect on other cognitive measures or behavioral symptoms in the study population.

Only one study concerning the use of DHA has been reported: using a randomized, double-blind design, Voigt and colleagues [91] tested the effect of 345 mg/day of DHA for four months upon 63 children (6–12 years old) with ADHD, all receiving maintenance therapy with stimulant medication. Despite blood phospholipid DHA content being increased in the active treatment group, there was no statistically-significant improvement in any ADHD symptoms compared to placebo. 

On the other hand, some investigations have provided more promising findings. In particular, interesting results have been obtained by the seven RCTs in which EPA and DHA were administered to ADHD patients and compared to placebo. 

Richardson and colleagues [84] published a pilot double-blind RCT investigating the efficacy of the combination of omega-3 and omega-6 fatty acids (daily dose of 186 mg of EPA, 480 mg of DHA, 864 mg of linolenic acid and 42 mg of arachidonic acid) vs. placebo in 41 children with ADHD-related symptoms and specific learning disabilities. ADHD-type symptoms were assessed using the Conners’s Teacher Rating Scale. After 12 weeks of treatment, mean scores for cognitive problems and general behavior improved more in the group treated with HUFAs than placebo. More recently, these findings were replicated by the same authors [85]. They conducted a larger double-blind placebo controlled RCT, including 117 children diagnosed with Developmental Coordination Disorder (DCD), which is characterized by deficit of motor functions and shows substantial overlap with ADHD in terms of difficulties with organizational skills and attention. The active treatment provided a high ratio of omega-3 and omega-6 fatty acids for three months. Despite no effects being reported on motor skills, HUFAs dietary supplementation led to improvement of reading, spelling and behavior in the treatment group compared to placebo. Subsequently, a one-way, uncontrolled crossover to active supplement followed for a further three months: similar changes were observed in the placebo-active group, while the original treatment group’s scores continued to improve. 

Sinn and Bryan (2008) on a large sample included 132 children aged 7–12 years with a diagnosis of ADHD [86]. Participants were enrolled in a 15-week double-blind controlled trial and were treated with 93 mg of EPA, 29 mg of DHA and 10 mg of gamma-linolenic acid daily, with or without a micronutrient supplement, and were compared to placebo. Children were not treated with ADHD medications. The results outlined that both groups treated with HUFAs improved more than placebo subjects in some core ADHD symptoms, such as inattention, hyperactivity and impulsivity. These results were confirmed by the same authors (Sinn and Bryan) [87] in a 15-week crossover study. 

Bélanger et al. (2009), in a Canadian double-blind, one-way, crossover randomized trial [88], measured the impact on ADHD of omega-3 HUFAs, using equivalent quantities of omega-6 HUFAs (sunflower oil) as the control condition. Thirty seven subjects were enrolled, but only 26 children succeeded in completing the study. Participants did not receive any other medication. They were divided into two groups (A and B) and participated in a 16-week, double-blind, one-way, crossover randomized study. In the first phase, Group A received the *n*-3 HUFA supplement, and Group B received placebo. During the second phase, Group B received the active *n*-3 HUFA supplement that was continued in Group A. In the first phase of the study (Weeks 0–15), a meaningful improvement in inattention and global ADHD symptoms emerged in patients who received omega-3 treatment (20 mg/kg/day–25 mg/kg/day of EPA and 8.5 mg/kg/day–10.5 mg/kg/day of DHA). These positive results were maintained during the second phase (the treatment crossover, Weeks 16–30) but did not reach in this phase statistical significance.

Kirby et al. [89] have assessed the effects of the administration of 0.8 g/day fish oil (including 400 mg of DHA and 56 mg of EPA/day) on 450 healthy school-children. After a period of 16 weeks, the plasma ratio omega-6/omega-3 was measured, and patients were submitted to a series of cognitive tests to assess IQ, reading, language and writing skills, attention, working memory, impulsivity and hyperactivity. This RCT showed a significant improvement in impulsivity, evaluated with the Matching Familiar Figures Task (MFFT), handwriting and attentional capacity, evaluated with the Computerized Penmanship Evaluation Tool (COCOM) and a possible protective effect of omega-3s on behavioral dysregulation, compared to placebo. 

In accordance with these findings, a six-month randomized double-blind controlled trial by Perera [90] indicated the efficacy of supplementation with a combined omega-3 and omega-6 preparation vs. placebo in 98 children with ADHD, refractory to methylphenidate treatment (all participants continued taking immediate-release methylphenidate during the study). In particular, omega-3 (600 mg/day) combined with omega-6 (360 mg/day) improved behavior and learning in restlessness, aggressiveness, completing work and academic performance, but not in inattention, impulsiveness and cooperation with parents and teachers.

A comprehensive meta-analysis conducted by Bloch et al. [96] of omega-3 fatty acids supplementation in children with ADHD found a small to modest effect size for EPA at high doses. Another recent meta-analysis conducted by Sonuga-Barke et al. [97] concluded that free fatty acids (omega-3 supplements, omega-6 supplements and both omega-3 and omega-6 supplements) produce small, but significant reductions in ADHD symptoms. Besides, it provides evidence to justify the use of omega-3 fatty acids for ADHD as a supplement to other empirically-supported therapies. Gillies et al. [98] suggested that the efficacy of omega-3 fatty acids is supported only in combination with omega-6 fatty acids. A recent review [99] found that randomized clinical trials with omega-3 HUFAs have reported small-to-modest effects in reducing symptoms of ADHD in children and that available findings need to be replicated in future investigations of nonpharmacological interventions in clinical practice (Table 4). 

### 2.7. Autism Spectrum Disorders

The efficacy of HUFAs as a treatment consideration has also been taken into account in other developmental disorders, such as autism spectrum disorder [100,101]. There is some evidence to suggest that autism may involve a cellular functional deficiency or imbalance of omega-3 [100,101,102]. Studies focused on the deficit in the concentration of HUFAs complexed to membrane phospholipids in children with autism showed controversial results. Vancassel and colleagues [102] examined the concentration of fatty acids in plasma in a population of children with autism and in another group of children with learning disabilities. This study reported a 23% reduction of DHA levels in the group with autism compared to the control group, a reduction in the erythrocytes membrane concentration of omega-3 and a concomitant increase in the levels of saturated fatty acids [100,101]. 

In addition, some evidence showed that omega-3 may improve the course of chronic inflammatory diseases [103], frequently associated with autism and potentially related to its pathophysiology [104]. 

Considering that data collected from uncontrolled studies are affected by severe limitations and that only three RCTs are available, evidence is insufficient at the moment to determine whether HUFAs are effective for autism spectrum disorder. Actually, only one study [105] supported HUFAs supplementation to treat autism-related symptoms, while the remaining did not find any positive effects [106,107].

The first RCT is a double-blind, randomized, placebo-controlled, pilot study, performed by Amminger in 13 children (aged 5–17 years) with autistic disorder [105]. After six weeks, the group treated with EPA (840 mg/day) and DHA (700 mg/day) showed an improvement of hyperactivity and stereotyped behaviors. 

The second double-blind, placebo-controlled trial was conducted by Voigt [106] and presented different conclusions. In particular, the authors did not show any improvement in core symptoms of autism after a dietary DHA supplementation of 200 mg/day for six months in a group of 48 children with autism. 

The third randomized, double-blind, placebo-controlled trial was conducted by Mankad et al. [107]. They designed a six-month trial of omega-3 fatty acid supplementation (1.5 g of EPA and DHA/day) in comparison with placebo in 38 children (2–5 years old) with autism. This study did not support the hypothesis that high dose supplementation of HUFAs in children with autism provides any efficacy in terms of improvement of core symptom domains or adaptive function.

Considering the scarcity of data in this field, we also reported the results from one case-report and one open-label study performed in patients with autism. The case report study [108] showed an improvement of symptoms in a child suffering from autism following treatment with EPA in the first time administered at a dose of 1 g/day, then at a dose of 3 g/day, for a period of four weeks. During the eight-month follow-up phase, the improvements were maintained. The open-label study [109] carried out on 20 autistic children reported a significant improvement of the disease after three months of treatment with omega-3, omega-6 and omega-9 at a dose of 1 g/day (1 g/day). 

To date, one systematic review [110] about the efficacy of HUFAs in the treatment of autism spectrum disorders is available. The conclusions showed that the omega-6/omega-3 fatty acid ratio’s alteration during early life can affect major processes in brain development and induces aberrant behavior. Thus, changes in dietary omega-6/omega-3 supplies may contribute to reducing the incidence of symptoms related to autism. So far, the studies are still few and provided limited results; therefore, further investigations on a larger population are required to draw conclusions in this field.

### 2.8. Aggression, Hostility and Impulsivity

In the last two decades, growing attention has been given to the potential role of omega-3 in clinical conditions characterized by high impulsivity, hostility and aggressive behaviors [5]. The discovery of low levels of EPA and DHA in the central nervous system of patients with impulsive-aggressive, self-harm and parasuicidal behaviors [111,112] has encouraged the investigators to perform trials on omega-3 implementation in this clinical population characterized by a high level of impulsivity and aggression with favorable results in terms of mood symptoms and control of impulsivity [113,114].

Two randomized, double-blind, placebo-controlled trials have been performed to analyze the effects of HUFA supplementation in populations without psychiatric diagnosis on aggression and impulsivity. Both showed positive results, but available data are limited by the heterogeneity of the study criteria. The first RCT [113] was conducted by Hamazaki in a group of 41 healthy controls (21–30 years old) monitoring the episodes of aggressive behaviors and cognitive functions during three months. Participants daily received 1.5–1.8 g of DHA or placebo. Aggression toward others significantly increased in the control group, while it decreased in the DHA supplement group. No significant differences were observed on cognitive functions. Another trial was conducted by Itomura et al. (2005) in 166 healthy school children 9–12 years old in order to investigate whether fatty acid nutrition may affect physical aggression [114]. Eighty-three children received a fish oil-fortified diet, providing a daily intake of 3.6 g of DHA and 0.84 of EPA for three months. Aggression against others and impulsivity were significantly decreased in the fish oil group, especially in girls. 

In a six-week RCT, Bradbury and colleagues (2004) [115] investigated the possible role of DHA in improving adaptation to perceived stress in a group of 47 healthy controls (18–60 years). They found a significant reduction in stress for both the fish oil (1.5 g/day of DHA) and the placebo groups, but the stress reduction for the fish oil group was significantly superior to that in the no-treatment controls. The fish oil group obtained a more substantial stress reduction than the olive oil group, but the differences between treatments did not reach statistical significance. 

### 2.9. Borderline Personality Disorder

The effects of omega-3 fatty acids supplementation has been studied in patients with personality disorders, who often show high levels of impulsive-behavioral dyscontrol and aggressiveness. Only two RCTs with a double-blind, randomized, placebo-controlled design have been conducted [116,117]. Both indicated the efficacy of HUFAs on borderline personality disorder (BPD) core symptoms, although they were different in diagnostic criteria, contemporary administration of conventional medications and the doses and ratios of omega-3 fatty acids.

Zanarini and Frankenburg [116] performed the first RCT in a group of 30 female patients with a diagnosis of BPD who were treated for eight weeks with 1 g/day of E-EPA or placebo and without any other psychotropic medication. The results showed a significant effect of E-EPA on aggressive behaviors measured with the Modified Overt Aggression Scale (MOAS) and on depressive symptoms assessed with the Montgomery-Åsberg Depression Rating Scale (MADRS) compared to placebo. In another study, published by Hallahan and colleagues [117], 49 patients with self-defeating behaviors (39 patients had received a diagnosis of BPD) were enrolled. Twenty-seven patients were randomly assigned to placebo, and 22 were treated with EPA at the dose of 1.2 g/day and DHA at the dose of 0.9 g/day for 12 weeks. Omega-3s were added to the standard psychiatric therapies. This RCT showed a significant improvement of affect (measured with the Beck Depression Inventory and the Hamilton for Depression Rating Scale (HDRS)), parasuicidal behaviors and stress reactivity in the treatment group. Aggressiveness, measured with the MOAS score, and impulsive behaviors, assessed with the Memory Delay Task, did not obtain a significant difference in the two groups. 

On the basis of this background, our research group performed a RCT in order to assess the efficacy and tolerability of omega-3 fatty acids in combination with valproic acid in a group of 43 BPD patients [118] who were randomly assigned to two treatments for twelve weeks: (1) valproic acid (800–1300 mg/day) (plasma range: 50–100 μg/mL); (2) EPA (1.2 g/day) and DHA (0.6 g/day) in combination with the same dose of valproic acid. Results indicated that the association of omega-3 fatty acids and valproate was effective in reducing the severity of characteristic BPD symptoms, measured with the Borderline Personality Disorder Severity Index (BPDSI), impulsive behavioral dyscontrol, assessed with the Barratt Impulsiveness Scale (BIS-11), outbursts of anger, evaluated with the item “outburst of anger” of the BPDSI, and self-mutilating conduct, measured with the Self-Harm Inventory (SHI) (Table 5).

### 2.10. Substance Dependence 

According to research, proinflammatory cytokines are responsible for the physical and psychological symptoms concomitant to craving, and EPA can neutralize these molecules’ toxic effects in the brain [75,119]. The neuroprotective effect of omega-3s on the production of serotonin and its action on the prefrontal cortex may also help with maintaining executive ability, both compromised during withdrawal and craving. Only two studies have been conducted to assess the efficacy of omega-3 fatty acid in the treatment of substance dependence [75,120]. The first study [75] evaluated the efficacy of EPA + DHA (3 g/day) for a period of three months on anxiety symptoms in addicted patients. The group treated with omega-3s showed a significant reduction in anxiety when compared to placebo. These results were replicated and confirmed by the following study performed by the same authors [120].

### 2.11. Anorexia Nervosa

Two studies, focused on the deficit in the concentration of HUFAs complexed to membrane phospholipids in patients with anorexia nervosa, showed similar results. Holman and colleagues [121] examined the concentration of fatty acids in plasma in a population of eight hospitalized anorexia nervosa fasting females compared to 19 healthy female adults <25 years old. Subjects with anorexia nervosa had deficiencies of essential fatty acids and showed decreased concentrations of total omega-6 and omega-3 acids, compared to the control group; this indicates compensatory changes in non-essential fatty acids and can lead to a consequent problem in terms of membrane structure and fluidity. Furthermore, Langan and Farrell [122] reported a concomitant significantly reduction of omega-3 and omega-6 in plasma phospholipids’ concentration in a group of 17 patients (16 females, one male) hospitalized for anorexia nervosa compared to 11 normal females. 

Only two trials have been conducted to assess the efficacy of omega-3 fatty acid in the treatment of anorexia nervosa. In the first pilot open label study, conducted by Ayton [123], seven patients between 13 and 22 years old with anorexia nervosa, restrictive subtype, received 1 g/day EPA in addition to standard treatment for three months. This small study showed a general improvement in sleep, mood, dry skin and constipation (measured with Weight 4 Height software, the Eating Disorder Inventory (EDI-2), the Morgan-Russell Average Outcome Scale (MRAOS), BDI-2, the Children’s Global Assessment Scale (CGA-S) and CGI-S. 

Negative results in this clinical population were reported by Barbarich and collaborators [124]. Twenty six subjects with anorexia nervosa (10 subjects were restricting-type; six subjects were restricting and purging only-type; and 10 subjects were binge eating/purging-type) participated in a six-month trial of fluoxetine (20–60 mg/die). Using a randomized, double-blind design, subjects were assigned to either nutritional supplements (600 mg of DHA and 180 mg of arachidonic acid daily, tryptophan, vitamins, minerals) or placebo. Patients were evaluated with: the Frost Multidimensional Perfectionism Scale (FMPS), the State-Trait Anxiety Inventory (STAI-Y) and Yale-Brown Obsessive Compulsive Scale (Y-BOCS). They were also weighed at weekly intervals for the first eight weeks, at two-week intervals for the following six weeks and at 4-week intervals for a further 12 weeks. There were no significant differences in weight gain per week between subjects treated with fluoxetine plus nutritional supplements vs. fluoxetine plus placebo. Moreover, there were no significant differences between groups in mean changes of anxiety or obsessive and compulsive symptoms. 

## 3. Adverse Effects

Omega-3 fatty acids did not induce serious adverse effects and were generally well tolerated: most common side effects reported in clinical trials were nausea and a fishy aftertaste, but they were mild and rarely induced discontinuation [125]. The Panel of The European Food Safety Authority (EFSA) concluded that the available data are insufficient to establish a tolerable daily intake (UL) of DHA, EPA and DPA individually or in combination, but the supplementation with EPA and DHA up to 5 g/day is not dangerous for the general population [126]. In particular, EPA and DHA are generally recognized as safe and well tolerated at dose up to 5 g/day in terms of bleeding risk, as pointed out by Yokoyama et al. [127] and Tanaka et al. [128]. In addition, doses up to 5 g/day, consumed for a maximum period of 12–16 weeks, do not significantly affect glucose regulation in both healthy and diabetic subjects [119,129,130,131] and do not increase infection risk by the activation of inappropriate inflammatory responses [132]. The intake of EPA and DHA at the same dose and up to 16 weeks does not induce alteration of lipid peroxidation and does not increase cardiovascular risk [133]. Combined intake of EPA and DHA at the dose of 2–6 g/day and intake of DHA at the dose of 2–4 g/die are responsible for an LDL concentration increase (3%), but do not affect cardiovascular risk. At last, an intake of EPA at the maximum dose of 4 g/day does not induce significant changes in LDL plasma levels [134].

## 4. Conclusions

In the last decade, the role of long-chain HUFAs in the treatment of several psychiatric diseases has gradually increased, as confirmed by the growing number of randomized controlled trials testing the efficacy of essential fatty acid, especially omega-3 HUFA, supplementation. Nevertheless, an overall consensus about their efficacy is still lacking, and the findings of most of trials are controversial and inconclusive. Differences in methods, including sample size, selection criteria, choice and dosage of fatty acids (i.e., EPA, or DHA, or a combination of the two, or the addition of omega-6 HUFAs) and the duration of supplementation often make results not comparable.

The main evidence for the efficacy of EPA and DHA has been obtained in mood disorders. In particular, omega-3 fatty acids seem to be useful in preventing and improving depressive symptoms at a low dose of 1 g/day; EPA seems to be more efficacious than DHA; patients with more severe depression showed greater treatment gains. However, due to the considerable heterogeneity of the investigations, additional large cohort studies and well-designed clinical trials are warranted. Concerning bipolar disorder, the results of systematic reviews and meta-analyses suggested a potential beneficial role of omega-3 fatty acids in addition to stable medications in treating depressive symptom at the approximate dose of 1–2 g/day, but did not support their use in attenuating mania. Furthermore, studies using a combination of EPA and DHA reported a statistically-significant improvement in symptoms of bipolar depression, whereas trials using a single compound did not. In schizophrenia, little evidence of a meaningful clinical effect was reported, and current data do not allow us either to refuse or support the use of omega-3 fatty acids in psychotic patients. However, adverse effects of antipsychotics, in particular metabolic abnormalities and extrapyramidal symptoms, may benefit from the addition of EPA or a combination of EPA and DHA. In ADHD disorder, several RCTs have been performed, but the main findings have reported small-to-modest effects of omega-3 HUFAs in reducing ADHD symptoms in children. Most promising results in this field have been reached by studies using EPA at high doses or the association of omega-3 and omega-6 fatty acids. Anyway, the relative efficacy of these agents was modest compared to the currently available pharmacotherapies. To date, a small number of clinical trials have explored the impact of HUFAs on impulsive and aggressive behaviors and a growing number of studies has been conducted in order to test the efficacy of these agents in borderline personality disorder. Results are encouraging, although this area of psychopathology needs to be explored in depth by future investigations. In autism spectrum disorders, only two RCTs with opposite results are available, whereas there is a substantial lack of data about the use of omega-3 fatty acids in anxiety disorders and obsessive-compulsive disorder. The majority of trials assessing patients with mood disorders did not investigate changes in anxiety symptoms, although anxiety is frequently associated with depression or mania. HUFAs were not found efficacious in treating eating disorders and substance use disorders. Concerning tolerability, RCTs considered in this review share a common finding: omega-3 fatty acids did not induce serious adverse effects. A survey of studies concluded that omega-3 are generally recognized as safe and well tolerated at doses up to 5 g/day for a maximum period of 12–16 weeks. Several authors have assessed the effects of omega-3 HUFA administration in terms of bleeding risk, glucose metabolism, lipid profile, infection risk and cardiovascular function with a general consensus about their harmlessness.

In summary, preliminary findings on omega-3 fatty acids in psychiatric populations allow us to consider these naturally-derived and well-tolerated psychotropic agents as a promising therapeutic tool. However, their efficacy in treating specific mental disorders or clusters of psychiatric symptoms is not sufficiently proven, as the findings from studies and reviews are too divergent to draw any conclusion.

## Figures and Tables

**Table 1:** Double-blind controlled trials of highly unsaturated fatty acids (HUFAs) as an add-on strategy in the treatment of schizophrenia. PANSS, Positive and Negative Syndrome Scale; E-EPA, ethyl-EPA.

Study	Drug and Dose	Sample	Treatment Duration	Results
Peet et al., 2001 [17]	EPA or DHA 2 g/day	45 patients	12 weeks	↓ psychotic symptoms measured with PANSS in the group treated with EPA
Peet et al., 2001 [17]	EPA 2 g/day	30 patients	12 weeks	↓ positive symptoms measured with PANSS
Peet and Horrobin, 2002 [19]	E-EPA 1–4 g/day	115 patients	12 weeks	↓ positive symptoms measured with PANSS, ↓ depressive symptoms
Jamilian et al., 2014 [20]	1 g/day	60 patients	8 weeks	↓ psychotic symptoms measured with PANSS
Fenton et al., 2001 [23]	ethyl-EPA 3 g/day	87 patients	16 weeks	no significant differences in positive, negative symptoms, mood or cognition
Berger et al., 2007 [24]	ethyl-EPA 2 g/day	69 patients	12 weeks	no efficacy on specific psychotic symptoms
Amminger et al., 2010 [15]	EPA 700 mg/day + DHA 480 mg/day	76 individuals “UHR”	12 weeks	↓ progression in psychosis in young UHR patients
Pawelzcyk et al., 2016 [21]	EPA + DHA 2.2 g/day	71 patients with FEP	26 weeks	↓ psychotic symptoms measured with PANSS ↓ depressive symptoms ↑ level of functioning
Bentsen et al., 2013 [22]	ethyl-EPA 2 g/day	99 patients	16 weeks	↓ impairment of the course of psychosis
Emsley et al., 2014 [18]	EPA 2 g/day + DHA 1 g/day + α-LA 300 mg/day	33 patients	2 years	relapse prevention of psychotic symptoms
Emsley et al., 2002 [29]	ethyl-EPA 3 g/day	40 patients	12 weeks	↓ positive symptoms and negative symptoms measured with PANSS

Abbreviations: EPA = eicosapentaenoic acid; DHA = docosahexaenoic acid; ethyl-EPA = ethyl-eicosapentaenoic acid; α-LA = α-lipoic acid; UHR = ultra-high-risk; FEP = first episode of psychosis; ↓ = decrease of; ↑ = increase of.

**Table 2:** Double-blind controlled trials of HUFAs in the treatment of depressive disorders. HDRS, Hamilton Depression Rating Scale; MADRS, Montgomery-Åsberg Depression Rating Scale.

Study	Dose and Method	Sample	Treatment Duration	Results
Peet and Horrobin, 2002 [35]	ethyl-EPA 1, 2 or 4 g/day add-on standard antidepressant treatment	70 patients resistant to antidepressant treatment	12 weeks	↓ depressive symptoms measured with HDRS, MADRS and BDI in the group treated with 1 g/day of HUFAs
Nemets et al., 2002 [40]	ethyl-EPA 2 g/day	20 patients	4 weeks	↓ depressive symptoms measured with HDRS from the second week of treatment
Nemets et al., 2006 [41]	ethyl-EPA 0.4 g/day + DHA 0.2 g/day	20 patients 6–12 years-old	16 weeks	↓ depressive symptoms measured with CDRS, CDI and CGI
Su et al., 2003 [36]	ethyl-EPA 4.4 g/day + DHA 2.2 g/day add-on existing antidepressant treatment	22 patients	8 weeks	↓ depressive symptoms measured with HDRS
Su et al., 2008 [42]	ethyl-EPA 2.2 g/day + DHA 1.2 g/day	36 pregnant patients	8 weeks	↓ depressive symptoms measured with HDRS, EPDS and BDI
Rondanelli et al., 2010, 2011 [43,44]	EPA 1.67 g/day + DHA 0.83 g/day add-on existing antidepressant treatment	46 elderly female residents in a nursing home	8 weeks	↓ depressive symptoms assessed with GDS, improvement of phospholipids fatty acids’ profile
Rees et al., 2008 [46]	ethyl-EPA 0.4 g/day + DHA 1.6 g/day	26 pregnant patients	6 weeks	no benefits on depressive symptoms
Freeman et al., 2008 [47]	EPA 1.1 g/day + DHA 0.8 g/day	59 women	8 weeks	no benefit on perinatal depressive symptoms
Lespérance et al., 2011 [37]	EPA 1.050 g/day + DHA 150 mg/day	432 patients with a major depressive episode	8 weeks	↓ depressive symptoms only for the patients without comorbid anxiety disorders
Mischoulon et al., 2008 [53]	DHA 1, 2, or 4 g/day	35 patients	12 weeks	measured in the group in treatment with 1 g/day of HUFAs
Jazayeri et al., 2008 [38]	EPA 1 g/day vs. fluoxetine 20 mg/day	60 patients	8 weeks	↓ depressive symptoms in both groups
Mischoulon et al., 2015 [54]	EPA 1 g/day or DHA 1 g/day	196 patients	8 weeks	EPA and DHA were not superior to placebo
Gertsik et al., 2012 [39]	EPA 1.8 g/day + DHA 0.4 g/day + other omega-3 fatty acids 0.2 g/day + citalopram vs. placebo + citalopram	42 patients	9 weeks	↓ depressive symptoms measured with HDRS
Carney et al., 2009 [55]	E-EPA (0.93 g/day) plus DHA (0.75 g/day) on depression in addition to sertraline	122 patients with major depression associated with coronary heart disease	10 weeks	EPA and DHA were not superior to placebo

Abbreviations: EPA = eicosapentaenoic acid; DHA = docosahexaenoic acid; ethyl-EPA = ethyl-eicosapentaenoic acid; CDRS = Childhood Depression Rating Scale; CDI = Childhood Depression Inventory; EPDS = Edinburgh Postnatal Depression Scale; GDS = Geriatric Depression Scale; ↓ = decrease of; ↑ = increase of.

**Table 3:** Double-blind controlled trials of HUFAs in the treatment of bipolar disorders (depressive and maniac phases).

Study	Dose and Method	Sample	Treatment Duration	Results
Stoll et al., 1999 [66]	EPA 6.2 g/day + DHA 3.4 g/day	30 patients	16 weeks	↓ depressive symptoms measured with HDRS
Frangou et al., 2006 [63]	ethyl-EPA 1 or 2 g/day add-on to stable psychotropic medications	75 patients	12 weeks	↓ depressive symptoms measured with HDRS
Chiu et al., 2003 [67]	EPA 4.4 g/day + DHA 2.4 g/day vs. placebo in addition to valproate 20 mg/kg/day	15 patients with acute mania	4 weeks	no significant differences between omega-3 fatty acids and placebo
Keck et al., 2006 [65]	EPA 6 g/day in addition to at least one mood stabilizer	121 patients with bipolar depression or rapid cycling bipolar disorder	4 months	no significant differences
Gracious et al., 2010 [68]	ALA in addition to psychotropic medication	children and adolescent with bipolar I or II disorder	16 weeks	significant improvement of overall symptom severity in comparison with placebo group
Murphy et al., 2012 [69]	omega-3 fatty acids plus cytidine, omega-3 fatty acid plus placebo or only placebo in addition to a mood stabilizer	45 patients with type I bipolar disorder	4 months	no benefits of omega-3 fatty acids on affective symptoms

Abbreviations: EPA = eicosapentaenoic acid; DHA = docosahexaenoic acid; ethyl-EPA = ethyl-eicosapentaenoic acid; ALA = α-linoleic acid; ↓ = decrease of; ↑ = increase of.

**Table 4:** Double-blind controlled trials of HUFAs in the treatment of ADHD.

Study	Dose and Method	Sample	Treatment Duration	Results
Voigt et al., 2001 [91]	DHA 345 mg/day vs. placebo; with ADHD medication	63 children (6–12 years old) with ADHD	4 months	no statistically-significant improvement in any ADHD symptoms compared to placebo
Richardson et al., 2002 [84]	EPA 186 mg·g/day + DHA 480 mg/die + linolenic acid 864 mg/die + arachidonic acid 42 mg/die vs. placebo	41 children with ADHD-like symptoms	12 weeks	mean scores for cognitive problems and general behavior improved more in the group treated with HUFAs than placebo
Stevens et al., 2003 [81]	DHA 480 mg/day + EPA 80 mg/day + arachidonic acid 40 mg/day + gamma-linolenic acid 96 mg/day vs. placebo; no ADHD medications	50 children with ADHD-like symptoms	4 months	no significant difference between active group and placebo was observed for any rating scale comparing patients who completed the trial
Hirayama et al., 2004 [92]	EPA 100 mg/die + DHA 500 mg/die vs. placebo; mostly without ADHD medications (only six subjects had been under medications)	40 children with ADHD	2 months	no evidence of the efficacy of omega-3 fatty acids compared to placebo
Johnson et al., 2009 [93]	EPA 558 mg/die + DHA 174 mg/die + gamma linoleic acid 60 mg/die vs. placebo; only one patient with ADHD medication	75 children and adolescents 8–18 year old with ADHD	3 months	no evidence of the efficacy of omega-3 fatty acids compared to placebo
Bélanger et al., 2009 [88]	EPA 20–25 mg/kg/die + DHA 8.5–10.5 mg/kg/day vs. placebo; no ADHD medications	26 children	16-week	improvement in inattention and global ADHD symptoms only in the first phase of the study (Weeks 0–15)
Milte et al., 2012 [94]	EPA-rich oil (providing EPA 1109 mg and DHA 108 mg), DHA-rich oil (providing EPA 264 mg and DHA 1032 mg) vs. an omega-6 HUFAs oil; no ADHD medications	90 children (7–12 year old) with ADHD	4 months	no statistically-significant differences between the two groups
Widenhorn-Müller et al., 2014 [95]	EPA 600 mg/die + DHA 120 mg/die; no ADHD medications	95 children (6–12 years) with ADHD	16 weeks	improved working memory function, but no effect on other cognitive measures or behavioral symptoms in the study population
Sinn and Bryan, 2008 [87]	EPA 93 mg/day + DHA 29 mg/day + gamma-linolenic acid 10 mg/day vs. placebo; no ADHD medications	132 children (7–12 years) with ADHD		improved in inattention, hyperactivity and impulsivity in most ADHD scales in parents’ reports; no improvement in teacher reports; limits: no ADHD diagnosis (reported ADHD symptoms)
Perera et al., 2012 [90]	omega-3 + omega-6 vs. placebo; with ADHD medications	98 children (6–12 years) with ADHD diagnosis	6 months	improved behavior and learning in restlessness, aggressiveness, completing work and academic performance, but not in inattention, impulsiveness and cooperation with parents and teachers
Kirby et al. [89]	DHA 400 mg/day + EPA 56 mg/day; no ADHD medications	450 healthy school-children	16 weeks	significant improvement in impulsivity, handwriting and attentional capacity and a possible protective effect of omega-3 on behavioral dysregulation, compared to placebo

Abbreviations: EPA = eicosapentaenoic acid; DHA = docosahexaenoic acid.

**Table 5:** Double-blind controlled trials of HUFAs in the treatment of impulsivity and borderline personality disorder.

Study	Drug and Dose	Sample	Treatment Duration	Results
Hamazaki et al., 1996 [113]	DHA 1.5–1.8 g/day	41 healthy controls (21–30 years)	3 months	↓ aggression
Bradbury et al. (2004) [115]	DHA 1.5 g/day	47 healthy controls (18–60 years)	6 weeks	↓ level of stress
Itomura et al. (2005) [114]	DHA 3.6 g/day + EPA 0.84 g/day	166 healthy controls (9–12 years)	3 months	↓ aggression, ↓ impulsivity
Zanarini and Frankenburg, 2003 [116]	EPA 1 g/day (with no standard psychiatric therapies)	30 BPD females	8 weeks	↓ aggression, ↓ depression
Hallahan et al., 2007 [117]	EPA 1.2 g/day + DHA 0.9 g/day (added to the standard psychiatric therapies)	49 patients with self-defeating behaviors (39 BPD patients)	12 weeks	↓ depression, ↓ parasuicidal behaviors, ↓ stress reactivity
Bellino et al., 2014 [118]	EPA (1.2 g/day) + DHA (0.6 g/day) in combination with valproic acid (800–1300 mg/day) vs. valproic acid (800–1300 mg/day) (plasma range: 50–100 μg/mL)	43 BPD patients	12 weeks	↓ severity of BPDSI, ↓ impulsive behavioral dyscontrol, ↓ anger, ↓ self-mutilating conduct

Abbreviations: EPA = eicosapentaenoic acid; DHA = docosahexaenoic acid; ethyl-EPA = ethyl-eicosapentaenoic acid; BPDSI = Borderline Personality Disorder Severity Index; ↓ = decrease of; ↑ = increase of.
